# Creating different global health futures: mapping the health research ecosystem and taking decolonial action

**DOI:** 10.1186/s12913-025-12566-3

**Published:** 2025-04-17

**Authors:** Nadia Tagoe, Seye Abimbola, Davide Bilardi, Dorcas Kamuya, Lucy Gilson, Kui Muraya, Sassy Molyneux, Caesar Atuire

**Affiliations:** 1 Kwame Nkrumah University of Science and Technology, Kumasi, Ghana; 2 University of Sydney, Sydney, Australia; 3 University of Oxford, Oxford, UK; 4 KEMRI-Wellcome Trust Research Programme, Kilifi, Kenya; 5 University of Cape Town, Cape Town, South Africa; 6 London School of Hygiene and Tropical Medicine, London, UK; 7 Independent Researcher, Nairobi, Kenya; 8 University of Ghana, Accra, Ghana

**Keywords:** Decolonisation, Knowledge systems, Funding, Knowledge production, Education, Uptake, Research practices, Tools

## Abstract

This paper promotes reflexive consideration of health research practices using a decolonisation lens. We propose both incremental and more radical action in five domains: knowledge production, funding and programmes, dissemination, uptake, and education and training. We suggest four steps towards transformation and share a reflexive tool to operationalise these steps.

## Introduction

Over the last few years, decolonisation has become an increasingly prominent topic in the global health literature, emphasising the need to challenge and change entrenched power asymmetries that have persisted as legacies of colonisation. Decolonisation questions deep-rooted assumptions and practices, and calls for structural change across the full spectrum of our global socio-economic, educational, health, governance, and financial systems [1]. Decolonisation involves identifying, challenging, and undoing the systems, structures, beliefs and narratives that perpetuate colonial relationships between and within countries and communities, and promoting self-determination by those whose voices and perspectives tend to be marginalised [2, 3]. Global health as a concept and practice is often perceived from diverse viewpoints with limited consensus on its definition or what it represents [4, 5]. In this paper, we adopt Koplan et al’s [6] definition which considers global health as “an area for study, research, and practice that places a priority on improving health and achieving health equity for all people worldwide”. Thus, calls for decolonisation in global health are a call to pay greater attention to systems and practices that perpetuate inequities and power asymmetries including racism, patriarchy, casteism, sexism, ageism, and nationalism [7–9].

Research is central to global health efforts as it contributes to our understanding of health and well-being, and informs policy and practice aimed at building and maintaining healthy populations [10, 11]. The need to address hegemony in research and knowledge systems has long been advanced in the literature, initiated well before the current emphasis on decolonisation in global health [12, 13]. We acknowledge that there are multiple dimensions of hegemony including social, political, economic, and cultural, that have implications on research processes such as the dominance of the English language and the strong emphasis on ‘evidence-based’ reasoning and ‘the scientific method’ in health research which may be foreign to many cultures [14–16]. The overarching point is that, addressing these unfair practices in health knowledge systems needs to be a crucial component of decolonisation in global health.

The goal of this paper is to promote reflection on our actions as diverse actors in the field of global health research using a decolonisation lens. We map out key constituents of the global health knowledge ecosystem and propose potential targeted action towards change. We hope that this mapping can contribute to reflection, discourse and research on the structures, actors, processes, and practices involved in health research, and commitment to possible actions for positive transformation in global health.

Our work, as a multidisciplinary Decolonisation and Global Health Research Exchange Network, has broadly focused on unpacking decolonisation debates in health research and broader knowledge practices to contribute to a research and action agenda for positive transformation in the field. We adopted critical reflection in this inquiry, recognising the value of this approach for collaborative inquiry, for research that examines implicit assumptions, and in providing a conceptual space for more liberal ways of thinking [17, 18]. Our reflections draw on the literature and on our diverse experiences across different disciplinary, cultural, and practice perspectives. Following conceptual discussions on what decolonisation embodied, we conducted bimonthly reflexive sessions over two years to examine our own and others’ understanding of how colonisation manifests in health research including in its structures, processes and practices, and the kind of praxis we envision.

To facilitate identification of colonial legacies in health research, and to move past discussions and make decoloniality practical, we recognised the need to delineate different components of global health processes for more critical scrutiny. We considered that without such an approach, the decoloniality discourse can be so sweeping and conceptual that it renders direct effective action complicated. Drawing from our practical experiences and knowledge, and the literature, we laid out the start-to-finish process for knowledge generation and use, and domains that feed into this process. We also catalogued some of the reflexive questions we consider as helpful for examining this process, and shared these in four in-person interactive international meetings for critique and enrichment by colleagues.

We acknowledge that we have our own biases and there are likely many other approaches that could contribute to the desired change. We also recognise the importance of multiple types of knowledge and ways of knowing [19–21], and that there are multi-dimensional complexities at play in decolonisation beyond the (often contested) binaries of high-income countries (HIC) vs low- and middle-income countries (LMIC), and Global North vs Global South [22, 23]. We remain open to multiple perspectives in our consideration of knowledge due to our belief that we do not necessarily have to agree on definitions but rather on shared and practical goals that are relevant to policy and practice [5].

## The incremental to radical continuum

As calls for decolonisation have gained traction, there have been pockets of change across the health research ecosystem, which can be termed incremental changes. Solidarity has been increasingly demonstrated through multi-country and multi-sectoral collaborations to address global health challenges [24–26]. Measures to address inequities and power asymmetries have been introduced in several funding, research, and publishing processes [27, 28]. For instance, the BMJ Global Health has mandated the inclusion of author reflexivity statements as part of manuscript submission requirements, which is intended to check exploitative research practices [27]. Capacity strengthening of less-resourced project partners has also become an essential component of most funded research projects [29, 30]. These positive changes are shifting practices and promoting equity and inclusion across the global health field and must be reinforced and expanded. Nonetheless, promoting equity, diversity and inclusion (EDI), while important, is not yet decolonisation. Giving others a seat at the table does not change how the table came to be, what the agenda on the table is and who it serves the most, how those previously seated got to be there, what informed who is being offered a seat, and whether those offered seats are free to fully participate [31, 32]. It is essential to address the deeper structures that govern and continue to (dis)empower voices at the table. While we embrace incremental approaches [33], we also recognise that more action along the spectrum towards radical approaches [34] will be critical to sustained change. We consider radical approaches as measures that interrogate and change the fundamental assumptions and practices in place. Combining incremental and more radical, fundamental measures is crucial as decolonisation requires internalised changes that affect thought and behaviour as well as structural and systemic changes that affect processes and practice.

It is crucial to ascertain whether current changes in the field, even if spread and scaled more widely, are far-reaching or deep-rooted enough to address the fundamental issues of coloniality. This can be done by first unpacking the concept of coloniality to illuminate its constituent themes. For instance, coloniality encapsulates, among other things, domination, imposition, prioritisation of some voices and delegitimization and silencing of others [35]. These processes continue to feature in global health practice to date, and Eurocentric thinking and modes of operation have remained dominant and largely unquestioned [16]. The result of this prevalent practice is that many actors’ agency has been suppressed or even eroded. The effects of coloniality are therefore too deeply ingrained to be addressed by changing procedures without challenging the deeper underpinnings. Decolonial action will require reversing existing dominance and promoting self-determination of actors that are often marginalised including some local researchers and research participants [3].

The deployment of incremental efforts is not a sufficiently effective process of decolonisation, particularly where these efforts are un-coordinated and fragmented. Further, focusing on incrementalism without the adoption of more radical measures risks those efforts being co-opted and undermined and ultimately enabling the preservation of the status quo [36]. Given that global health is birthed from the supremacy of countries, regions, disciplines, institutions and populations [37], there is a need for a bolder approach to decolonisation that systematically challenges the *raison d’etre* of existing structures and processes leading to systemic changes across the entire global health chain [38]. Beyond thinking about what can be reformed, we also advocate for thinking about what needs to be abolished and entirely reconsidered. It may be worth dismantling some existing global health structures to rebuild new ones with different assumptions which will provide opportunity to imagine and create a different future. Acknowledging that there are many ways of being, knowing and doing, as a starting point, should help broaden how global health is perceived and operationalised [37, 39, 40]. We recognise that this approach is neither a simple nor linear process with guaranteed results. However, we anticipate that embracing both the incremental and the radical will create opportunities for deeper change.

## Mapping of the global health research ecosystem

We identified five key interlinked health research domains of the global health research ecosystem that play critical roles in the generation, dissemination and use of knowledge for improved health outcomes. These are: 1) **knowledge production** which cover the research cycle from conceptualization through data and results generation; 2) **research funding and programmes** which refer to the processes and practices used in designing and funding global health research and capacity strengthening programmes; 3) **knowledge dissemination** which encompasses choice of dissemination platforms and target audiences, authorship decisions and publishing practices of journals; 4) **knowledge uptake** which takes into account the decisions on how research outputs are prioritized for utilization by relevant stakeholders; and 5) **education and training** which consider processes for determining the content and approaches used in educational institutions and training of health researchers. It will be valuable to evaluate and seek to transform each of these domains. It is important to note that the outlined domains should be seen as a start to more comprehensive mapping rather than a prescriptive or exhaustive mapping. Additionally, the domains, while discretely mapped out, are tightly interlinked, with practices in one domain consequentially influencing or being replicated in other domains.

### Knowledge production

In global health, knowledge production processes have been widely accepted as pre-determined scientifically proven steps which form the basis for judging what qualifies as research, its quality, and the value accorded to the outputs. As a result, overt and covert hierarchies exist across research methodologies, among different health disciplines, between and within institutions, among researchers, within research teams, and between researchers and research participants. These hierarchies are upheld by this technoscientific approach although it is only one approach to knowledge generation emerging from the Eurocentric culture [16]. Research priorities are often determined by actors in the research space who are considered privileged based on their discipline or role and whether they are based in HICs or LMICs [41–43]. Decolonisation of the research process may require giving greater prominence to research methods that adopt more holistic perspectives and facilitate learning about local priorities and grounded issues. Such methods include accessing the tacit knowledge of local actors rather than strict adherence to research procedures linked to a Eurocentric technoscientific approach to knowledge production [16, 44, 45]. We acknowledge that the term local and what is considered local knowledge are complex and often contested, with knowledge in any setting having been influenced by several epistemologies over time. However, in promoting decoloniality, it is essential that the priorities and philosophy deferred to when generating knowledge within any context should be owned by those who the research seeks to benefit. Incremental approaches currently in use, such as in the Health Policy and Systems Research field, include varying forms of participatory research where studies are undertaken with rather than on participants [46–48], anthropological research [49, 50], co-production [51, 52], and two-eyed seeing which involves integrating indigenous and technoscientific knowledge [53, 54]. Some of these strategies include flattening research hierarchies, empowering indigenous knowledge actors, and mainstreaming diverse knowledge systems and ways of knowing. More radical approaches include redefining the point of departure for health research through for example valuing and using indigenous knowledge as the starting point of a body of knowledge (rather than as filling a perceived gap in existing knowledge), and interrogating how a system functions so it can be improved through action learning approaches [55, 56] (rather than through seeking answers to pre-conceived questions). Decolonized knowledge production processes require that no group of persons, whether internal or external to the local system, maintains the hegemony of the health episteme or the right to determine knowledge needed by others [57, 58].

### Research funding and programmes

Eligibility requirements in global health funding calls, composition of funding committees, and funding decisions that favour HIC applicants have been called out repeatedly [59, 60]. Similarly, funded programmes are often skewed in multiple ways – for example, agenda setting by HIC actors for LMIC-oriented programmes, leadership and conceptual roles held by HIC partners while LMIC partners are given more operational roles, and mismatched resource and benefit sharing [60, 61]. The power asymmetry arising from resource imbalance permeates all the other domains. Funders are therefore often targeted for criticism because of the power they wield and the downstream effect of their decisions. For instance, the Global Fund is an important funder of HIV/AIDS, malaria, and tuberculosis programmes, but only two of the 22 voting board members represent the Africa region [62]; and this is despite Africa shouldering a disproportionate percentage of the global burden of these diseases – HIV (73%), TB (25%) and malaria (94%) [63, 64]. We recognise efforts by some funders to increasingly ensure that funding and programme implementation decisions are increasingly made by actors in the regions most affected. For example, the Wellcome Trust are increasingly engaging reviewers from the contexts of planned programmes to assess grant applications. To promote more fundamental change, global health funding practices and research agenda setting should ensure that the research goals and processes centre the needs and priorities of those with lived experience of research contexts and study foci. Funders could adopt beneficiary-initiated bottom-up research programme design without pre-determined focus areas such as infectious diseases, and etic-driven research as bases for programmes. This requires the design phase to be supported and considered as part of the overall programme. The short-term project approach to most research initiatives often limits such constructive and locally driven processes and more sustainable capacity strengthening due to requirements to demonstrate performance with tangible indicators within a few years [65]. Promoting self-determination also means local governments, corporate bodies and philanthropic organisations must take up the responsibility of allocating resources to local research priorities. Increased local funding is essential to balancing the power dynamics in the research space [66, 67].

### Knowledge dissemination

Addressing coloniality means unearthing and strengthening diverse knowledge dissemination systems and centring and amplifying indigenous voices and marginalised knowers. Similar to knowledge production processes, research dissemination practices are subject to a hierarchical ranking of dissemination channels that values peer-reviewed publications over other forms of outputs and publications. Due to promotion criteria in academic institutions, publishing in some journals is still valued over others irrespective of the intended audience for the research findings [68, 69]. While it should be intuitive for local issues to be directed to local audiences, somehow, it has become a norm to publish local research in foreign journals. To serve the foreign gaze [68], local realities are often massaged and sometimes even repressed due to gatekeeping strategies adopted by publishers. In recent years, there have been efforts to enforce equitable authorships and diversify editorial boards and increasing criticism for publication metrics and journal ranking. For example, the Lancet has implemented the practice of rejecting papers with data from Africa that fail to acknowledge African collaborators [70]. Health Policy and Planning has recently established authorship criteria that require the inclusion of at least one co-author based in the countries where the research was undertaken [71]. Bolder decolonial action will require going beyond author diversity to epistemological diversity by valuing and publishing tacit and experiential knowledge from local realities [40]. Additionally, standards for journals could include regional and linguistic foci to not only promote local dissemination platforms for local audiences but to decentralise the perceived knowledge hubs. These actions will thrive if collective efforts are made to strengthen and amplify local and regional dissemination platforms. Decolonising knowledge dissemination may also mean going further to question the primacy of journals in research dissemination and epistemic power structures that have made global health more of an academic activity than an endeavour primarily aimed at street-level transformation. It means ensuring accessibility of the knowledge, language, and context by those who are most affected by it and those who are best placed to drive change. Currently, such practices are disincentivised in most research institutions as career progression and promotion criteria are heavily dependent on peer-reviewed publication.

### Knowledge uptake

The COVID-19 pandemic has illuminated the inequities enrooted in knowledge uptake processes where access to research outputs such as vaccines portray discriminatory practices [72]. Certain types of health knowledge or products are prioritized for uptake, and decisions on who benefits from new knowledge or products are often weighted more by HIC organisational preferences and nationalistic interests than by the needs on the ground [35, 73]. Further, the top-down approach to research described above undermines knowledge uptake, where funders, foreign partners, and academics determine the research needed more than the knowledge users, and where research is considered a means of generating publications for academic career progression more than for providing solutions to local health needs.

There have long been calls to ensure funded research captures the needs of the most relevant beneficiaries to enhance the usefulness of the outputs. The 10/90 gap, which indicates that less than 10% of global spending on health research is devoted to conditions that account for 90% of the global disease burden, powerfully demonstrates this mismatch [74]. Similarly, growing concerns have been raised about the corporatization of health [75–77]. Given that global health research outputs are public goods, the decisions and processes that generate and make these goods accessible to users must be driven by equity and social justice concerns. More importantly, systemic politico-economic and market drivers of health delivery systems that determine issues such as restricted intellectual property rights of research outputs need to be addressed to fully realize equitable access [78].

### Education and training

The strengthening, growth and continuity of the global health field is hinged on training of both existing and future actors in the field. Many global health training programmes, particularly in university settings and largely HIC-based have been underpinned and shaped by colonial systems and perspectives [79, 80]. Indeed, the origins of global health are traced back to tropical medicine which primarily sought to address the health challenges of Europeans based in colonized territories due to colonial governance, explorations, and wars, and to preserve the labour force in extractive industries [34, 37, 81, 82]. Tropical medicine was therefore a core element of political and economic pursuits of HICs [83]. Applying a decolonial approach to the transformation of education and training systems will require first examining the definition of and assumptions underpinning global health. Several authors have highlighted flaws of global health as currently conceptualized and practiced including its distant pose over local engagement, lack of contextuality, and its dependence on the definer [23, 84, 85]. The myriads of definitions and perspectives together with the contestations are important to highlight in teaching global health. Further, the nuances of the historical, political and socio-economic contexts within which global health is embedded and the resulting epistemic supremacy of some actors in global health needs to be examined [80]. For instance, the use of global university ranking criteria across various contexts have been widely critiqued as inappropriate and inequitable [86, 87]. In recent times, there have been efforts among global health institutions in both the Global North and the Global South to enhance EDI in education and to interrogate and rethink curricula. For example, the Imperial College London has designed and piloted a new model in their BSc Global Health curriculum to explicitly incorporate firsthand sharing of lived experience by LMICs actors [88]. Decolonial approaches in education will require capturing multiple contexts and worldviews in curriculum design, inclusive teaching methodologies and more effective means of assessing institutional performance [86, 89]. Transforming this domain is essential to avoid inadvertent reproduction of the very system we are seeking to change.

### Five domains, one goal

Exploring decolonisation in health research across the five domains highlights the intersection of several concepts including epistemic injustice, power and privilege, inequitable partnerships, and social injustice [21, 90, 91]. These concepts have been drawn upon to critique knowledge practices such as institutionalizing epistemological dominance of biomedical and quantitative knowledge over other forms of knowledge by journals, funders, and other stakeholders, effectively silencing those other forms of knowledge. Clearly, decolonisation efforts must consider these complex interactions across multiple domains and adopt a systemic and systematic approach to attain positive transformation. Decolonisation is not limited to Global North and Global South asymmetries. Hence, to make significant strides, actors from both the Global North and Global South will be required to accept and act on their responsibilities.

## Steps towards change

**Fig. 1:**
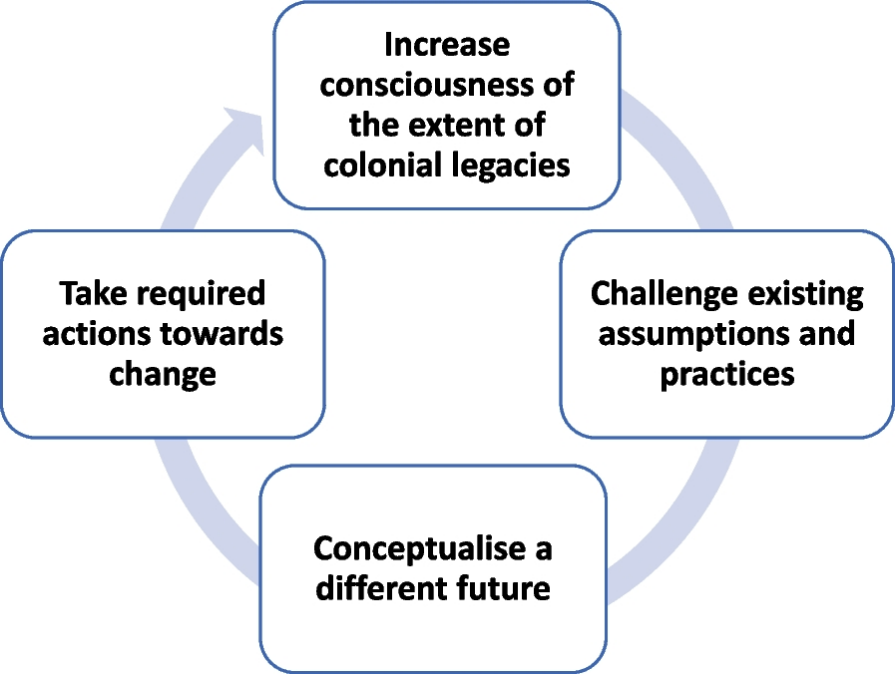
Steps towards decolonisation

We support others in arguing that we must combine current incremental measures across the five domains with more radical solutions to decolonise knowledge practices. We propose four steps for consideration as a potential pathway to transformation within each identified domain and more generally: increasing consciousness of the extent of colonial legacies, challenging existing health research assumptions and practices, conceptualising a different future, and taking the required actions that moves us towards that different desired future (Fig. 1). These steps are not meant to be distinct and sequential but rather form a continuous iterative process that can help drive decolonisation.

### Increase consciousness of the extent of colonial legacies

Decolonisation calls for raised consciousness of the colonial foundations on which global health practice rests and an awareness of the indicators and root causes of a colonised system [89]. This requires a conscious engagement with the decolonisation discourse to enhance understanding and enable its recognition by actors across various contexts. A critical part of consciousness should be directed at oneself, wherever one is in the world, through reflexivity and increasing awareness of one’s positionality, thinking and practices in each of the domains. As health researchers and practitioners with varying backgrounds, we should be continuously reflecting on our different training and experiences, how our thinking has been shaped, and how the current global health system continues to shape our perspectives and actions. This should increase our awareness of the drivers of power, justice, equity, and ethics, and how these influence our interactions, activities and outputs in our fields of practice. Reflexive exercises that enable analyses and understanding of our power - privilege and marginalisation – should support increased awareness and changing of mindsets.

### Challenge existing assumptions and practices

It is crucial that assumptions and practices in the field are continuously questioned and challenged, such as what kind of knowledge is considered ‘valid’, whose views really count, and who the research is really serving. Deliberate interrogation of both formal and informal processes and approaches including unwritten rules is required for deeper learning and to identify sites for potential structural change, where spaces are created to allow for new thinking and action.

### Conceptualize different futures

Critical analyses of assumptions and practices pave the way for conceptualising new and different futures for the relevant areas of operation and influence. The ability to visualize alternatives that represent a departure from the colonial elements that shaped the existing system is an important part of this process. It is crucial to continuously bear the principles that undergird (de)coloniality in mind to inform our thinking and choices as individuals, teams or institutions, and to enable the creation of a different future. This is a delicate process that needs to be carefully and collectively monitored and stewarded to avoid a new future designed solely by old actors wearing new hats. At the same time, the process needs to embrace a pluriversal approach that recognizes multiple viewpoints and a more inclusive and shared search for mutually enriching solutions.

### Take the required actions towards change

The imagined future(s) should serve as a frame of reference for facilitating new perspectives that reflect in action. New learning from the earlier steps should drive action at individual, institutional and systemic levels that are both incremental and radical. Sustainable change will require a wide range of actions affecting perceptions, assumptions, behaviour, engagement, structures, processes and practices. Such action can influence change across the knowledge generation and use spectrum including teaching, research and its processes, programme design and implementation, dissemination and publication, grant applications, and review of proposals and manuscripts.

While many actors desire to take such actions, translating the desire into practical steps in day-to-day health research processes is often seen as a challenge. Good practices to offer conceptual frameworks to enhance reflexivity in the global health spaces have been designed and promoted [92]. To operationalise the recommended steps for change, we propose a reflexive tool that can guide individuals and institutions in interrogating the status quo and identifying potential levers of change (Table 1). The tool presents sample questions that actors can build on and utilise in examining individual and institutional assumptions and practices in order to guide action on being, thinking and doing in each domain towards positive change.

**Table 1:** A reflexive tool for guiding decolonial action of individuals and institutions

**Domain**	**Reflexivity Questions for Individuals**	**Reflexivity Questions for Institutions**
1) Knowledge production	Which voices do I capture in determining research needs and concepts for research programmes and which voices are left out? Which ideals form the basis for determining knowledge gaps I seek to address, where do those ideals stem from and who do the ideals serve best? What do local systems consider as knowledge needs to improve their functionality? How best can I capture those needs? What types of knowledge do I value and which standards do I apply in determining this? Which types of knowledge for health have been silenced, de-emphasized, or demonised? Which actors do I consider as legitimate knowers? Why are certain knowers deemed unqualified or their knowledge less relevant? What methodologies for collecting data do I consider as acceptable or unacceptable and who determined that they are? How can I identify and leverage the multiple ways of knowing in the local context? What are the indigenous ways of knowing and approaches to generating new knowledge? Have I prioritised foreign approaches to research over local approaches and why? How can I rectify this if I have?	What types of knowledge production processes are considered acceptable to the institution, and what is the basis for this choice? Have we invested in identifying and developing indigenous and context-specific knowledge production processes beyond the status quo? Have actors within the local context but outside academic fields been considered as legitimate and valuable research team members? How can this be done? How best can power asymmetries in research teams that favour capacitated and more-resourced actors be addressed?
2) Funding and programmes	Which stakeholders do I consider as eligible members of research teams? Which stakeholders have I sidelined? What has informed these criteria? Whose voices drive the agenda for my research programmes? How do local actors (doers and thinkers) in health practices within the context feature in my health research programmes? Which types of knowledge generation activities and sources do I prioritise in allocating resources?	Why are some types of knowledge generation approaches considered more valuable and eligible for funding compared to other approaches? How can local processes be prioritised? Why are academic experts considered as preferred project leaders who are eligible as grant holders? How can criteria for project leaders and grant holders be made more inclusive? How can diverse types of knowledge production processes be considered as fundable programmes? How can LMIC team members lead all aspects of research programmes for LMIC-relevant research? How can more LMIC than HIC actors lead decision-making processes for programme design and funding?
3) Knowledge dissemination	What can I do differently in conducting and disseminating my research to serve the purposes of the population most affected by the research more than just the scientific community? What range of dissemination channels do I employ to make my research more accessible? Who benefits most from these channels? How can the gatekeeping rules and metrics used by research publishers be more equitable? How accountable am I to research beneficiaries and what requirements can ensure greater accountability? Do I value foreign platforms for disseminating locally relevant knowledge over local platforms? Why or why not? If yes, what can I do to rectify this situation?	Why are foreign platforms for disseminating locally relevant knowledge valued over local platforms? What can be done to rectify this anomaly? How can journals be made more accessible to the beneficiaries who are most affected by it? How can the gatekeeping rules and metrics used by research publishers be more equitable? What can be done to strengthen the locally/regionally focused dissemination platforms (including journals and conferences) to the required quality? What policies can be established (including for promotion) to build and value indigenous platforms for showcasing research outputs? How can funders influence where and how locally relevant research outputs are published to reach the populations that are most affected?
4) Knowledge uptake	To what extent are the needs and priorities of the end users of research prioritised in the agenda setting and development of my research? How can I make my research outputs accessible (including dissemination platform and language used) to end users to promote uptake?	How can research uptake systems be changed to address inequitable access to research outputs for enhanced health outcomes?
5) Education and training	How is my teaching and practice a reflection of the colonial perspective inculcated through my training? How context-specific is the focus, content and materials that I use in training others? What value do I place on the lived experience in my teaching content? How do I seek out the lived experience to highlight when teaching?	How can curricula inherited from colonial educational systems be substituted with context-specific content that value local perspectives? How are curricula and teaching and assessment approaches guided by the heterogeneity of the audience?

## Looking ahead

In this paper, we have sought to promote essential reflection and action towards transforming health research practices using a decolonisation lens. Our aim is to stimulate thinking and fresh perspectives in global health research in ways that upend the status quo and facilitate sustainable change. Global health knowledge systems have conventionally been optimised to follow pre-specified research procedures which tend to marginalise untapped forms of knowledge and ways of knowing. We hope that these considerations will promote health research that centres on its core essence, which is learning that improving systems and behaviours rather than stringent procedures which often discredit other forms of epistemologies and practice.

We recognise that while there are opportunities, there are also constraints within the current system that can hinder or lengthen the transformative process. Propositions for realising different futures may not always be realized as envisaged; however, we hope that they will trigger processes that lead to positive change. As proposed, both incremental stepwise actions and radical transformational shifts are important approaches to change. Similarly, addressing the hardware (including processes, structures, and content) and software (including power, culture and values) of the research eco-system need to be leveraged at both global and local levels.

Finally, it is important to remember lessons from decolonisation of physical territories as we seek to decolonise global health and the wider context within which the field exists. Decolonisation is not just about ensuring actor sovereignty and inclusion as these alone do not necessarily bring about the equity and justice desired. Decolonisation is not only about changing governance and operational structures and processes but rethinking ideologies and practices which are much harder to dismantle. Again, we should be careful not to undo or endanger the positive outcomes through practices such as partnerships and solidarity. Although such approaches may have been systemically altered by the broader structures within which they are practiced, they are innately positive and valuable in tackling health challenges in our interconnected world. Overall, we recognise that a different global health practice is required by us all if we are to attain health equity more widely, starting with new ways of being, thinking and doing until a critical mass results in a transformed field.

## Data Availability

No datasets were generated or analysed during the current study.

## Abbreviations

AIDS: Acquired ImmunoDeficiency Syndrome
COVID-19: COronaVIrus Disease of 2019
EDI: Equity Diversity and Inclusion
HIC: High-Income Country
HIV: Human Immunodeficiency Virus
LMIC: Low and Middle-Income Country
TB: Tuberculosis
